# Soil inoculum identity and rate jointly steer microbiomes and plant communities in the field

**DOI:** 10.1038/s43705-022-00144-1

**Published:** 2022-07-26

**Authors:** Xu Han, Yingbin Li, Yuhui Li, Xiaofang Du, Bing Li, Qi Li, T. Martijn Bezemer

**Affiliations:** 1 Erguna Forest-Steppe Ecotone Research Station, Institute of Applied Ecology, Chinese Academy of Sciences, Shenyang, 110016 China; 2 Forestry College, Beihua University, Jilin, 132013 China; 3 University of Chinese Academy of Sciences, Beijing, 100049 China; 4 Department of Terrestrial Ecology, Netherlands Institute of Ecology (NIOO- KNAW), Wageningen 6700 AB, Wageningen, The Netherlands; 5 Institute of Biology, Above-Belowground Interactions Group, Leiden University, P.O. Box 9505, 2300 RA Leiden, The Netherlands

**Keywords:** Biodiversity, Food webs

## Abstract

Inoculation with soil from different ecosystems can induce changes in plant and soil communities and promote the restoration of degraded ecosystems. However, it is unknown how such inoculations influence the plant and soil communities, how much inoculum is needed, and whether inocula collected from similar ecosystems will steer soil and plant communities in different directions. We conducted a three-year soil inoculation experiment at a degraded grassland and used two different soil inocula both from grasslands with three inoculation rates. We measured the development of the soil and plant communities over a period of three years. Our results show that soil inoculation steers the soil microbiome and plant communities at the inoculated site into different directions and these effects were stronger with higher amount of soil used to inoculate. Network analyses showed that inoculation with upland meadow soil introduced more genera occupying the central position in the biotic network and resulted in more complex networks in the soil than inoculation with meadow steppe soil. Our findings emphasize that there are specific effects of donor soil on soil microbiomes as well as plant communities and that the direction and speed of development depend on the origin and the amount of soil inoculum used. Our findings have important implications for the restoration of biodiversity and ecosystem functioning in degraded grassland ecosystems.

## Introduction

Grasslands cover about 40% of the earth’s surface, providing a large number of ecosystem services [1, 2] including food and water supply, carbon storage and climate change mitigation [3]. However, the intensification of land use has resulted in a worldwide degradation of species-rich forage grasslands and undermined their ability to support biodiversity and ecosystem functioning [4–6]. Grassland degradation is widespread over the world, with 49% of global grassland area being degraded [5]. Soil organisms are essential for soil organic matter decomposition and nutrient cycling, and thereby can affect plant production and community dynamics [7–9]. Recent studies have shown that inoculation of soil collected from a late successional field can alter soil microbial communities and facilitate the establishment and growth of late-successional plant species through the plant-soil interactions at the recipient site [10–13]. The interplay between plant and soil communities can lead to a positive effect on the dynamic of the plant community [14, 15]. For example, Wubs and co-workers found that the composition of both soil and the plant communities in a former arable field was steered towards a predefined natural community when using soil inoculum collected from a semi-restored grassland and heathland [12]. There is compelling evidence that inoculation with soil from two distinctly different ecosystems can induce various changes in the soil and in plants communities in the field [16]. However, a major outstanding question is whether inoculation with soil originating from underneath different plant communities but from a similar ecosystem will also lead to differential development of the soil and plant communities and ultimately the entire ecosystem at the recipient site.

In previous soil inoculation studies, greatly varying amounts of donor soil have been used ranging from 0.016 cm up to 40 cm layers of donor surface soil [17, 18], while little research focused on the effect of inoculation amount of soil. We may expect that there is a positive relationship between the amount of soil that is used and the establishment of soil biota and plants. Results from a controlled pot experiment showed that there was a positive effect between the amount of inoculated soil and plant biomass [19]. However, in the field, and over longer time periods, it is also possible that due to positive feedbacks between the soil and plant communities, inoculation with a small amount of donor soil will have a similar effect on the soil microbiome and plant communities as higher amounts of donor soil [12, 16]. How the amount of donor soil that is used for inoculation influences the establishment of the soil microbiome and plant communities in the field is still not known. In restoration projects, the donor soil typically originates from a nature area, and collecting this soil will inevitably cause some destruction of the donor area. Hence it is essential to better understand the minimum amount of soil inoculum required for successful restoration to avoid over-exploitation of the donor area.

**Fig. 1:**
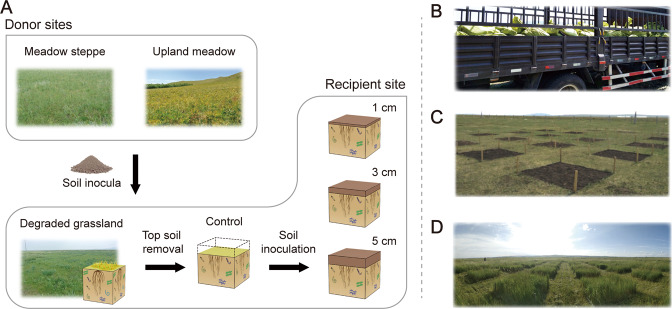
Graphical illustration of the experimental design. **A** Three replicate meadow steppes and upland meadows were selected as donor sites. At each donor location, the soil of the top 10 cm was excavated, homogenized and transported to the degraded grassland. The experiment was set up with three fully replicated blocks. In each block, one plot without inoculation was also established. We established 19 experimental plots of 2 × 2 m^2^ (2 soil types × 3 replicate sites × 3 inoculum amounts + control) in each block and a total of 57 plots. **B** The transfer of soil inoculum in 2018 to the field; **C** the experimental field site after inoculation in 2018; **D** and three years later, in 2021.

We conducted a soil inoculation experiment in a degraded grassland and measured the development of the soil microbiome, soil nematodes and the plant communities over a period of three years following soil inoculation. We used two donor grasslands: a meadow steppe and an upland meadow, and inoculated three different amounts of donor soil in experimental plots at the degraded site (Fig. 1). We hypothesized that inoculation with the different donor grassland soils would steer soil microbiomes and plant communities towards the donor soil microbiomes and plant communities (H1). We further hypothesized that inoculation with upland meadow soil will lead to a better restoration than meadow steppe through higher biodiversity in forest-steppe ecotone (H2). Finally we hypothesized that the effects of soil inoculation will be accelerated with increasing amounts of donor soil used to inoculate the field plots (H3).

## Methods

### Experimental design

The experiment was carried out at a degraded grassland near the Erguna Forest-Steppe Ecotone Research Station of the Chinese Academy of Sciences (50°10′46.1″N, 119°22′56.4″E). The mean annual temperature is −2.4 °C with minimum value in January (−28 °C) and maximum value in July (19.1 °C). The mean annual precipitation is 361.6 mm mainly concentrated in summer and autumn. The texture of the surface soil (0–10 cm) is 47% sand and 51% clay and silt [20].

In 2018, a degraded grassland was selected as recipient site. Three replicates of meadow steppe and upland meadow were selected as donor sites. The soil type is classified as chernozems in degraded grassland and meadow steppe, chernozems or kastanozems in upland meadow based on the World Reference Base for Soil Resources (WRB) [21]. In the experiment, we inoculated soil from two donor grassland ecosystems, a nearby meadow steppe and an upland meadow with a distance of about 20 km. The meadow steppe was a grassland with moderate disturbance of human activity due to mowing each year dominated by xerophytic grasses. The upland meadow belongs to the forest-steppe ecotone with little disturbance dominated by mesophytic forbs. Three replicate sites were set for each donor site with about a 1 km distance between each site. From 21 May to 29 May, at each donor location, we removed all plants and the top 10 cm of soil was then excavated and homogenized by spade manually. Soil inoculum was transported to the degraded grassland by truck from meadow steppe and upland meadow, respectively. The top 5 cm of soil from the degraded grassland was removed (top soil removal treatment to facilitate restoration [12, 19, 22]) by creating cuboid blocks (2 × 2 × 0.05 m^3^) using spade and the soil was dumped outside the experimental area. At the degraded grassland, the soil was inoculated at three inoculation amounts: 1 cm, 3 cm and 5 cm (equals to 10 L m^−2^, 30 L m^−2^ and 50 L m^−2^) with a rate of 1.34 g cm^−3^ for meadow steppe and 1.13 g cm^−3^ for upland meadow, respectively (based on the corresponding bulk density). Soil inocula was weighed and compacted to the predefined depth to keep similar bulk density. The experiment was set up with three replicated blocks. In each block, one plot with top soil removal but without inoculation was also established. In each block, we established 19 experimental plots (2 soil types × 3 replicate sites × 3 inoculum amounts + control) and a total of 57 plots were established. Each plot was 2 × 2 m^2^ in size with 2 m wide paths between them. The central 1 × 1 m^2^ quadrat was used for vegetation recording and small quadrats (0.25 × 0.25 m^2^) were set adjacent to the central quadrat at different locations each year for destructive sampling of soil and plant properties. In 2018, all 57 samples were analyzed for soil properties, and plant and nematode community composition. For bacterial and fungal sequencing, we homogenized soil between blocks (except control) so a total of 21 samples were analyzed in the first year. In 2019 and 2020, all 57 samples were sequenced for microbial communities. In 2020 we also collected soil samples at the donor sites and recorded the biomass and vegetation composition to compare the restoration effect in the third year.

### Soil sampling and analysis

In August of 2018, 2019 and 2020, within each plot, ten soil cores (2.5 cm diameter, 10 cm deep) from one of the adjacent quadrats (0.25 × 0.25 m^2^) were collected and pooled as a composite sample for each plot. After gentle homogenization and removal of roots, 10 g fresh weight of soil was immediately stored at −80 °C for DNA analysis. About 100 g fresh weight of soil was kept in a plastic bag at 4 °C for nematode community analysis, and the remaining soil was sieved through a 2 mm mesh, air dried and analyzed for soil physicochemical properties. Soil moisture was determined by oven-drying subsamples at 105 °C for 24 h. A soil: water (1: 2.5) mixture was used for measuring soil pH with a glass electrode. Total carbon (TC) and total nitrogen (TN) content in each sample were determined using a TruSpec CN Elemental Analyzer (Leco Corporation, USA). Total phosphorus (TP) was determined by the method of molybdenum-antimony colorimetric using a spectrophotometer (Shimadzu Inc., Kyoto) [23].

### DNA extraction and amplicon sequencing

We extracted total soil DNA from 0.5 g soil using the FastDNA^TM^ Spin Kit (MP Biomedicals, USA), followed the manufacturer’s protocol. Bacterial 16 S rRNA genes (V4) were amplified using the primers 515 F (5′- GTGCCAGCMGCCGCGG-3′) and 907 R (5′-CCGTCAATTCMTTTRAGTTT-3′) [24], and fungal ribosomal internal transcribed spacers (ITS2) were amplified using the primers ITS86F (5′-GTGAATCATCGAATCTTTGAA-3′) and ITS4R (5′-TCCTCCGCTTATTGATATGC-3′) [25]. PCR reactions were conducted with 10 ng of extracted soil DNA as template per 20 μl PCR reaction which contained 4 μl 5 x FastPfu buffer, 2 μl 2.5 mM dNTPs, 0.4 μl FastPfu Polymerase, 0.2 μl BSA, 1.8 μl ddH20 and 0.8 μl of each forward and reverse primers. PCR cycling conditions were set for 95 °C for 3 min, followed by 27 cycles for bacteria and 35 cycles for fungi of denaturation at 95 °C for 30 s, annealing at 55 °C for 30 s, extension at 72 °C for 45 s and a final extension of 72 °C for 10 min. Amplicons were sequenced on the MiSeq PE300 platform (Illumina, San Diego, America) with PhiX spike-in as a quality and calibration control. Sequences were merged with FLASH v1.2.11 [26], barcode and primer removed with USEARCH v10.0 [27], quality filtered using FASTP v0.19.6 [28], dereplicated and clustered to OTUs with UPARSE v7.0.1090 according to 97% similarity [29], taxonomic annotated with the Ribosomal Database Project (RDP) classifier v2.11 [30]. For bacteria, we assigned the sequences to taxonomic groups using the SILVA 138 database [31] and we assigned fungi sequences to taxonomic groups using the UNITE 8.0 database [32]. For all following calculations, OTUs that occur in less than 3 samples and with relative abundances <0.01% were disregarded. To account for differences in sequencing depth, we rarefied the bacteria and fungi to 19,067 and 59,331 reads, respectively. The raw sequencing data was deposited in China National Microbiology Data Center (Accession: NMDC10017881).

### Nematode community analysis

Soil nematodes were extracted from 100 g of fresh soil according to a modified cotton-wool filter method [33, 34], with soil solution passed through different sieves (250 µm and 38 µm). We collected the nematode suspension after standing for 24 h and fixed each sample in 4% formaldehyde solution. We counted the total number of nematodes through a stereoscopic microscope (Leica stereo microscopy MZ 12.5, Germany) and at least 100 individuals were identified to genus level through a microscope (OLYMPUS BX51T 12P01, Japan) [35–37].

### Plant community analysis

In August of 2018, 2019 and 2020, species richness (number of species) and cover of each plant species were recorded in the 1 × 1 m^2^ central quadrat of each plot. The cover was represented by estimating the percentage of projected area of all plant species within the central quadrat. The aboveground vegetation was harvested by clipping all biomass at 1 cm from the soil level in one of the adjacent quadrats in each plot. In the third year, belowground biomass was also recorded. Three soil cores (5 cm diameter, 10 cm deep) inside the adjacent quadrat were collected from each plot and weighed, and roots were cleaned with water and a 2 mm sieve. All aboveground and belowground biomass were oven-dried at 60 °C to constant weight and dry biomass was recorded.

Plant species were classified as annual or perennial according to the Flora of China [38]. The ratio of perennial cover to total cover was calculated to reflect the level of grassland restoration [39]. From the plant species database, we further classified indicator species for degenerated sites, meadow steppe and upland meadow based on the Flora of China [38]. *Leymus chinensis* was considered as the most important restoration target species at the middle stages of restoration [40]. *Artemisia frigida*, *Cleistogenes squarrosa*, *Carex duriuscula* and *Potentilla acaulis* were selected as indicator species for degenerated sites. *Leymus chinensis* and xerophytic grasses (*Stipa capillata* and *Koeleria cristata*) were selected as meadow steppe target species. *L. chinensis* and mesophytic forbs (*Sanguisorba officinalis*, *Artemisia tanacetifolia*, *Vicia sepium*, *Trifolium lupinaster*, *Lathyrus quinquenervius*, *Galium verum* and *Carex pediformis*) were selected as upland meadow target species [41–43].

### Data analysis

All statistical analyses were carried out in R version 3.6.1. Composition of the vegetation was analyzed based on the relative plant cover (the percentage of plant cover). We analyzed univariate response data using linear mixed models, with separate models for soil physicochemical properties, soil biota and plant community data. First, we assessed the overall effect of year, soil type, inoculum amount and their interactions by linear mixed models with repeated measures, with site replicate and block as random factors. Second, we assessed the differences between all inocula and control treatment each year using Post hoc Scheffe tests (glht function in the “multcomp” package). Three linear mixed models, for each year, were constructed respectively with block as random factors. Linear mixed models were constructed by lmer (in the “lme4” package) function [44].

For 2020, we tested the effect of soil inoculation by calculating the RR (response ratio) between treatments and control, donor meadow steppe or donor upland meadow by

$${{{{{{{\mathrm{RR}}}}}}}} = \frac{{X_t - X_c}}{{X_c}}$$

Where X_t_ is the value of variables (soil and plant traits) in soil inoculation treatments. X_c_ is the value of corresponded variables of control, donor meadow steppe or donor upland meadow in the same block or same site replicates. Linear mixed models were used to test the soil type and amount effect on RR with soil type, amount and interaction as fixed effects, with block and site replicates as random factors.

To visualize the effect of year, soil type, inoculum amount and their interactions on soil microbiomes, nematodes and plant communities, we performed unconstrained principle coordinates analysis (PCoA) and permutational analysis of variance (PERMANOVA). Plant cover was Hellinger-transformed before this analysis. Separate permutational analysis with different years were conducted to test the year effect on soil organisms and plant communities. To examine to what extent the composition of the soil microbial and nematode communities in the inoculated plots resembled those of the donor site, we conducted PCoA with the third-year data from the experimental plots together with the donor sites. Ordination analyses were performed using the R package “phyloseq” based on Bray-Curtis dissimilarities and permutational analysis of variance using the functions adonis2 in the “vegan” package [45, 46].

We subsequently compared the similarity between inoculated plots and control/donor sites. We calculated the pair-wise similarity between each treatment to control, donor meadow steppe and donor upland meadow by 1–Bray-Curtis dissimilarity. We also assessed soil inoculation effects on similarity using a linear mixed models, soil type, inoculum amount and interaction as fixed effects, block and site replicate as random factor.

Soil inoculation will introduce diverse soil fauna due to differences in local conditions. These genera may show different effects on soil and plant communities. We agglomerate OTUs into genera to have the bacteria, fungi and nematode at the same taxonomic level. We identified the significantly increased genera (relative abundance) in the meadow steppe and upland meadow inoculation treatment compared with control plots. These genera were considered to be directly introduced by soil inoculation or indirectly promoted by inoculation. The genera were confirmed by both indicator species (in the “indicspecies” package) and LRT (likelihood ratio tests at a false discovery rate corrected value of *P* < 0.05 using Benjamini and Hochberg method, in the “edgeR” package) analysis [47–49].

We constructed co-occurrence networks by spearman rank correlations between all pairs of bacteria, fungi and nematode genera, significant correlations (*r* > 0.6 and *P* < 0.001) were shown. The relative percentages of different biota were centered log-ratio transformed before analysis to avoid unit-sum constraint [50–52]. All networks were visualized with the Fruchterman-Reingold layout with 10^4^ permutations in “igraph”. The network complexity was calculated as linkage density. We generated random networks and compared these to our networks to test whether the networks were random using the erdos.renyi.game function in “igraph” [53]. Microbial taxa that frequently co-occur with other taxa in microbial co-occurrence networks are thought to be ecologically important which can influence the soil structure and functioning of soil communities. We selected the top 10 classified central genera (also termed as key genera) with the highest node degrees of co-occurrence and tested the Spearman correlations between their relative abundance and inoculation amount, soil and plant traits [49, 54–56].

## Results

### Inoculum identity and amount steer belowground communities

**Fig. 2:**
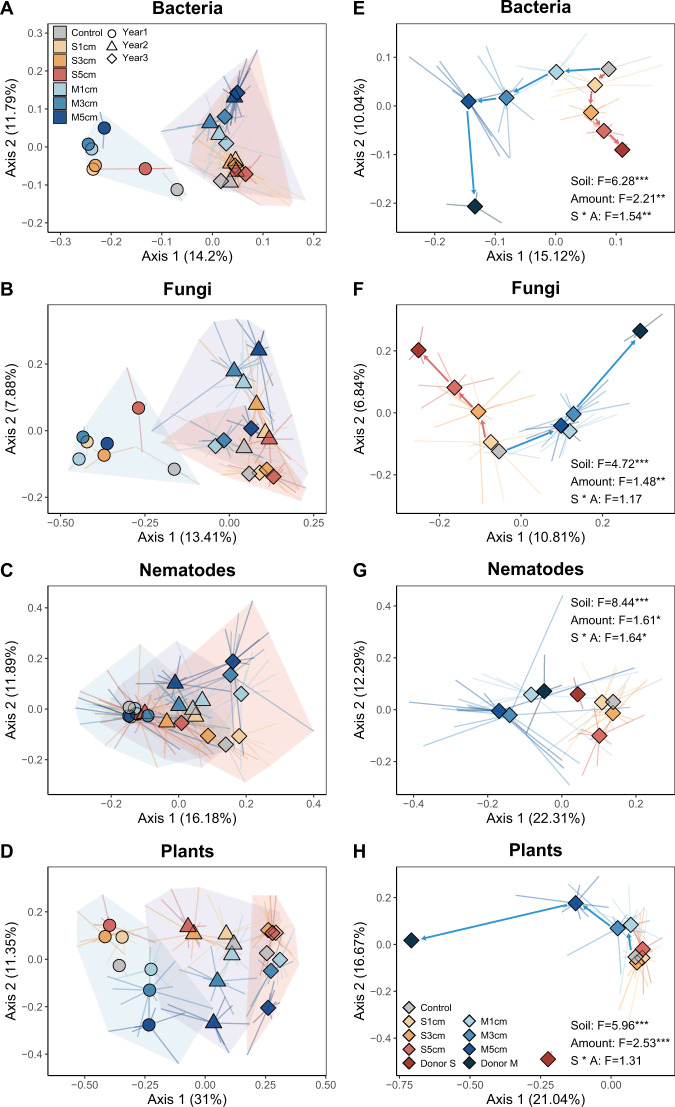
Effects of soil inoculation on soil bacteria, fungi and nematodes, and plant communities. Separate PCoA ordinations using Bray-Curtis distance are presented for three years (**A**, **B**, **C** and **D**) and for the third year in comparison to the donor (**E**, **F**, **G** and **H**). Percentage of variation given on each axis refers to the explained fraction of total variation. The colors depict soil inoculation treatments: Control, plot without inoculation; S1cm, meadow steppe soil inoculation with 1 cm depth; S3cm, meadow steppe soil inoculation with 3 cm depth; S5cm, meadow steppe soil inoculation with 5 cm depth; M1cm, upland meadow soil inoculation with 1 cm depth; M3cm, upland meadow soil inoculation with 3 cm depth; M5cm, upland meadow soil inoculation with 5 cm depth; Donor S, donor meadow steppe; Donor M, donor upland meadow. The different shapes and shaded areas jointly represent plots in different years: circle, for the first year; triangle, for the second year and rhombus, for the third year. The endpoint of each line represented the replicated sample points in each treatment. The result of PERMANOVA for three years were showed in Table 1 and for the third year were listed above, *P < 0.05; **P < 0.01; ***P < 0.001.

The identity of the donor soil and inoculation amount both significantly affected the soil microbiomes and nematode communities in the degraded grassland (Fig. 2A–C; Table 1). Already after three years, the bacterial and fungal communities had diverged towards those of the donor soil and inoculum amount strengthened this divergence (Fig. 2D–F). Soil inoculation effects on the nematode community were limited in the first year but significant in the second and third year (Table S1). Richness of bacterial and fungal communities were also affected by soil inoculation (Figs. S1, S2). Increasing the amount of inoculum had a positive effect on fungal richness (F = 15.26, *P* < 0.001) but a negative effect on bacterial richness (F = 3.21, *P* = 0.044). Additionally, richness of the bacterial community was lower in soils inoculated with upland meadow soil than that with meadow steppe soil (F = 5.6, *P* = 0.02). Together, these results show that donor soils originating from a similar ecosystem can steer the soil communities at the degraded grassland towards the specific donor community but also, for the first time that in the field, this is accelerated when increasing amounts of inoculum are used.

**Table 1:** Effects of year, soil type, inoculum amount and their interactions on soil and plant communities (Adonis test).

	Bacteria			Fungi			Nematodes			Plants		
	R^2^	F	*P*	R^2^	F	*P*	R^2^	F	*P*	R^2^	F	*P*
Year	0.087	13.4	<0.001	0.082	11.89	<0.001	0.097	18.24	<0.001	0.262	71.64	<0.001
Soil	0.068	10.53	<0.001	0.051	7.33	<0.001	0.029	5.41	<0.001	0.071	19.48	<0.001
Amount	0.046	3.55	<0.001	0.029	2.09	<0.001	0.02	1.91	0.008	0.049	6.7	<0.001
Year * Soil	0.007	1.1	0.305	0.008	1.13	0.237	0.022	4.07	<0.001	0.017	4.67	<0.001
Year * Amount	0.012	0.89	0.656	0.009	0.63	0.998	0.013	1.21	0.11	0.016	2.17	0.011
Soil * Amount	0.027	2.07	<0.001	0.021	1.51	0.016	0.014	1.35	0.131	0.03	4.18	<0.001
Year * Soil * Amount	0.011	0.84	0.786	0.01	0.69	0.992	0.01	0.97	0.417	0.007	1.01	0.432

### Soil inoculum identity and amount steer plant communities

**Fig. 3:**
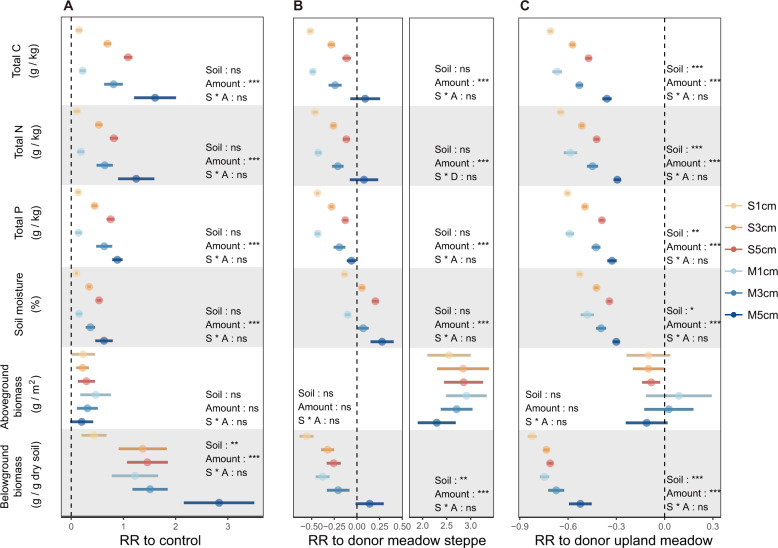
Response ratio of soil and plant traits to control and donor sites. Response ratio of soil and plant traits to control (**A**), donor meadow steppe (**B**) and donor upland meadow (**C**). Error bars represent ±S.E. The category definitions are as follows: S1cm, meadow steppe soil inoculation with 1 cm depth; S3cm, meadow steppe soil inoculation with 3 cm depth; S5cm, meadow steppe soil inoculation with 5 cm depth; M1cm, upland meadow soil inoculation with 1 cm depth; M3cm, upland meadow soil inoculation with 3 cm depth; M5cm, upland meadow soil inoculation with 5 cm depth. *P < 0.05; **P < 0.01; ***P < 0.001.

Soil inoculation also significantly affected plant community composition (Fig. 2D; Table 1) and steered the composition of plant communities towards the specific grassland donor sites (Fig. 2H). The proportional cover of perennials increased over time (F = 168.82, *P* = 0.006) and was higher in plots inoculated with upland meadow soil than in plots that received meadow steppe soil (F = 92.57, *P* < 0.001) (Fig. S3). With increasing amount of inoculum, species richness of the plant community also increased and plant species richness was higher in upland meadow than in meadow steppe inoculated plots (Fig. S2). When compared to the uninoculated control, soil inoculation suppressed the growth of degenerate indicator species, and increased the relative cover and abundance of the target species *L. chinensis* (Fig. S3). The total cover of all target species increased over time (meadow steppe: F = 92.4, *P* = 0.011; upland meadow: F = 79.37, *P* = 0.012) but this was not affected by inoculum amount (Fig. S4). More upland meadow target species colonized in plots inoculated with upland meadow soil (F = 15.62, *P* < 0.001) but meadow steppe target species did not colonize more successfully in plots inoculated with meadow steppe soil than in control plots (Fig. S4). In the third year, belowground (root) biomass was significantly higher in inoculated than in control plots (F = 11.74, *P* < 0.01), and this effect increased with increasing amount of inoculum used (F = 11.60, *P* < 0.001) but no significant effect was observed for aboveground biomass (soil: F = 0.41, *P* = 0.53; amount: F = 0.26, *P* = 0.77; soil × amount: F = 0.58, *P* = 0.56). Concentrations of soil nutrients and soil moisture also increased with increasing inoculum amount (Fig. 3,  Fig. S5).

### Similarity to the control and the donor sites

**Fig. 4:**
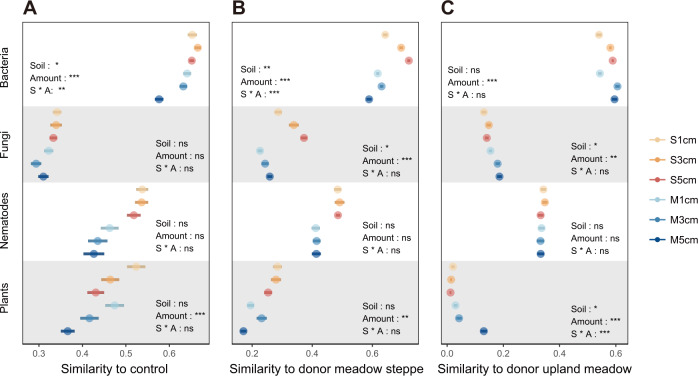
Similarity of soil biotic and plant communities to control and donor sites. Similarity between soil inoculation treatments to control (**A**) or donor treatments (**B**, **C**). Error bars represent ±S.E. The category definitions are as follows: S1cm, meadow steppe soil inoculation with 1 cm depth; S3cm, meadow steppe soil inoculation with 3 cm depth; S5cm, meadow steppe soil inoculation with 5 cm depth; M1cm, upland meadow soil inoculation with 1 cm depth; M3cm, upland meadow soil inoculation with 3 cm depth; M5cm, upland meadow soil inoculation with 5 cm depth. *P < 0.05; **P < 0.01; ***P < 0.001.

We further analyzed the effectiveness of inoculation by comparing the similarity of the soil and plant communities of inoculated and uninoculated plots and the similarity of inoculated plots to the two donor sites in the third year (Fig. 4). With increasing amount of soil inoculum, inoculated plots became more dissimilar from the control (uninoculated) plots for bacterial communities and for plants. Inoculation with meadow steppe soil increased the similarity to donor meadow steppe more than inoculation with upland meadow for bacterial and fungal communities and a positive effect of inoculation amount was found for bacterial and fungal communities. Similarly, inoculation with upland meadow soil resulted in higher similarity to the upland meadow donor site for fungi and plants and a positive effect of the amount of inoculum was found for bacterial, fungal and plant communities.

### Soil networks

**Fig. 5:**
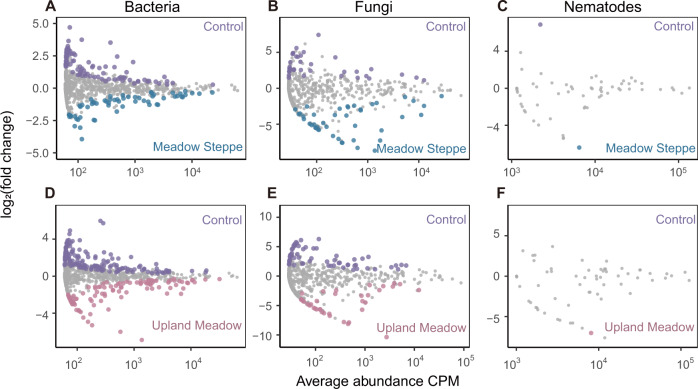
Soil inoculation effects on specific sets of bacteria, fungi and nematodes. Abundance patterns of meadow steppe soil inoculation treatments in bacterial (**A**), fungal (**B**) and nematode (**C**) communities (blue) and of upland meadow soil inoculation treatments in bacterial (**D**), fungal (**E**) and nematode (**F**) communities (pink) in comparison with the control (purple). The X-axis shows average abundance CPM (counts per million), and the Y-axis shows log_2_ (fold change) (control relative to meadow steppe inoculation or upland meadow soil inoculation). Blue, pink and purple points showed specific genera in meadow steppe, upland meadow and control respectively, and non-differential genera are colored in gray (likelihood ratio test, *P* < 0.05, FDR corrected).

**Fig. 6:**
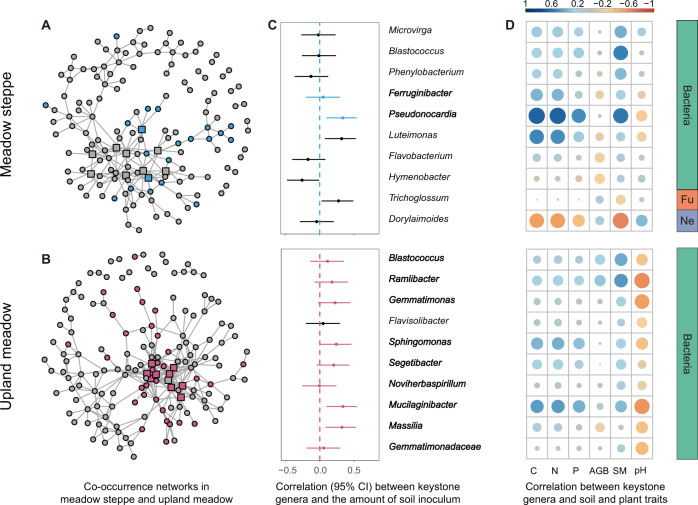
Network analysis and increased genera after inoculation with different donor soils. Co-occurrence networks at genus level based on meadow steppe (**A**) and upland meadow (**B**) soil inoculation. Correlation between the relative abundance of 10 central genera with the highest node degree and soil inoculum amount (**C**) and soil and plant traits (**D**). Blue points (in **A**) and pink points (in **B**) indicated the genera significantly increased after inoculation meadow steppes and upland meadow soil than control plot. Square in networks (in **A** and **B**) and bold genera names (in **C**) indicated the genera significantly increased after soil inoculation. C: soil total carbon, N: soil total nitrogen, P: soil total phosphorus, AGB: aboveground biomass, SM: soil moisture, Ne: nematodes, Fu, fungi.

Soil genera with higher relative abundance in the inoculation plots than in the control were selected (Fig. 5). These genera may be directly introduced by soil inoculation or indirectly promoted following inoculation. To confirm whether these genera play an important role in the soil community, we explored the distributions of bacterial, fungal and nematode communities in co-occurrence networks (Fig. 6A, B). The ten central genera in the network (higher node degree values, i.e. related with more other genera) were selected (Fig. 6C). Most of those genera significantly correlated with soil and plant traits (Fig. 6D). We also found that nine out of these ten central genera increased after inoculation with upland meadow soil, and they also positively correlated with the amount of soil inoculum. However, only two genera increased after inoculation with meadow steppe soil occupying the central position in the biotic network. Network complexity was also higher after inoculation with upland meadow (2.12) than after inoculation with meadow steppe soil (1.58).

## Discussion

This soil inoculation experiment at a degraded grassland with two different soil inocula and three inoculation rates shows that in the experimental plots soil and plant communities diverged and that these effects were stronger when more soil was used for inoculation. Moreover, inoculation with upland meadow soil introduced more central genera that may play important role in the soil networks than inoculation with meadow steppe soil. These findings emphasize that there are specific effects of donor soil on the developing soil and plant communities, these effects depend on the identity of the donor even when comparing two grassland donors.

The importance of the role of soil organisms in the restoration of plant communities is becoming widely acknowledged [57, 58]. Soil inoculation can be used to promote the colonization of soil organisms at the start of restoration. This study now shows that inoculation with soil communities that originate from different donor grasslands steers soil microbiomes as well as plant communities of the inoculated degraded grassland site into different directions. This result highlights the importance of selecting the correct donor site in restoration projects. In our study, inoculation with both meadow steppe and upland meadow soil led to a higher similarity to the specific donor but plant community inoculation with upland meadow soil caused much stronger effects than inoculation with meadow steppe soil. More target plant species from the upland meadow colonized the inoculated site after inoculation with upland meadow soil, while colonization of meadow steppe target plant species after inoculation with meadow steppe soil was less successful. These findings suggest that there are distinct differences in colonization or establishment success of soil microorganisms and plant among different donor soils. Interestingly, these findings also show that differences in the initial composition of the whole soil inoculum, can alter interactions in the soil networks at the degraded grassland and this could suggest that the choice of donor can have important consequences for the composition of soil communities and the functioning of the entire ecosystem that is restored. A previous soil inoculation experiment sowed standardized seed mixtures in all soils to disentangle the effects of soil inoculation via the microbial community and via the introduction of seeds and root pieces. That study showed that soil inoculation steered plant community into the direction of the donor sites also when the effects of introduction of plant propagules were excluded [12]. Field studies should be designed that compare the effects of only introducing seed mixtures (e.g. sown vs unsown plots), only soil inoculation (e.g. by sowing seed mixtures in inoculated and non-inoculated plots) and both seeds and soil communities (e.g. in control vs inoculated plots, as in the current experiment). Hence, to what extend the inoculum-specific changes in the plant communities in the current study were directly caused by introducing different species of plants (e.g. as seeds or root pieces) in the inoculated soil or whether this was due to different rates of establishment of the plants mediated by the altered soil community requires further work.

An important result from this study is that the changes in the soil and plant communities and the similarity to the donor sites increased almost linearly with the amount of inoculum used. These findings support our third hypothesis that the effect of soil inoculation on soil microbiomes and plant communities is accelerated with increasing amounts of soil used for inoculation. In an experiment with trees, stronger inoculation effects on the height and diameter of ectomycorrhizal tree species were observed when higher amounts of soil inoculum (1.5 L per seeding than 0.3 L per seeding) were used [59]. It is likely that with the increasing amount of soil, the density of seeds, root pieces and soil organisms linearly increased and that this results in increased establishment of the introduced organisms [60]. However, it is important to note that for many plant species, germination of seeds from the seedbank only occurs at the soil surface [12] and in our experiment for all inoculum amounts, the soil surface was covered with a layer of inoculated soil. Inoculum amount can also influence the establishment and survival of the introduced soil biota. It is likely that the introduced soil community survives and establishes best in the local habitat and that increasing the amount of inoculated soil at the recipient site increased resemblance to the original habitat conditions by preventing the effects of desiccation and by better resembling the original abiotic conditions such as nutrient availability and pH [16, 18]. A better functioning new soil community could, in turn, be important for the establishment of target plant species. Many functions that soil biota provide are density dependent [19] and the density of soil organisms should be sufficiently high to avoid mortality and inbreeding depression [16, 18]. In this study, upland meadow soil inoculation also introduced some central taxa which may play important roles by determining microbiome functioning, such as, *Massilia* and *Mucilaginibacter*. The abundance of those genera positively correlated with the amount of inoculum and with the soil and plant traits. These bacteria can promote plant growth at the early successional stages, and in soil carbon and nitrogen cycling [61, 62], which may, in turn, influence the biogeochemical processes at the degraded grassland. Future experiments are needed to assess whether these genera directly or indirectly influence the other community members in the soil microbiome and the performance of plant communities. If some central taxa can be further identified, artificially constructing microbial communities containing these taxa for soil inoculation may be an effective way to promote the restoration of degraded grasslands in the future.

Over the first three years following soil inoculation, the restoration of the degraded grassland soil and plant communities (i.e. the similarity to the donor sites) improved considerably. Importantly, the effects that we observed were not strongest immediately after inoculation and then declined, but instead the effects increased over time [59]. For example, soil inoculation effects on the nematode community were limited in the first year but significant in the second and third year. This clearly shows that the effects of inoculation were not remnants of what was left behind in the inoculated soil, but instead, that soil inoculation initiated the development of new soil and plant communities at the degraded site. Nematodes occur at different positions in the soil food web, and our results show that also higher trophic levels of the soil food web are influenced by soil inoculation, already two to three years after inoculation, even though organisms at these trophic levels are typically slow-growing and have low dispersal [63, 64]. Soil inoculation to steer degraded grassland towards a donor site follows the concept of close-to-nature restoration which takes account of natural processes, especially ecological processes mediated by soil organisms, to achieve sustainable restoration [65]. Our results show that whole soil inoculation is a promising method by which not only microbiomes but entire soil food webs can be reshaped rapidly in the field and as such can be a close-to-nature restoration tool in ecosystem restoration projects.

Overall, the experiment highlights that soil inoculation can promote restoration of degraded ecosystems but that the way and direction the ecosystem develops after inoculation depend strongly on the origin of the inoculum and the amount of inoculum. Via soil inoculation we created new soil and plant communities in the field and these new aboveground and belowground communities will influence each other interactively. A recent study in a species rich grassland on a former arable field showed that it can take up to a decade before such interactions become apparent [59]. Therefore, long-term experiments are needed to determine how the new aboveground and belowground communities will interact and how this will change the functioning of these grassland ecosystems and the composition of species in those ecosystems. Selecting grasslands with different degrees of degradation to conduct long-term experiments with different amounts of soil inoculum and determining the relationship between grassland degradation degree and soil inoculum amount can also provide a reference for evaluating the inoculum amount in practice. Moreover, long-term measurements are needed to better determine whether the ecosystems that received small amounts of inoculum, in the longer term, will exhibit a similar but delayed pattern of development as the ecosystems that received higher amounts of inoculum, or whether instead the former will become similar to the uninoculated controls over time. Such long-term data will also reveal whether and for how long the ecosystems that were inoculated with different donor soils will continue to develop into different communities (e.g. via different above-belowground interaction loops), or whether they will converge over time.

## Data and materials availability

All data needed to evaluate the conclusions in the paper are presented in the paper and/or the Supplementary Materials. Additional data related to this paper may be requested from the corresponding author.

## Supplementary information

Supplementary Information
