# Supplementary material for: Soil inoculum identity and rate jointly steer microbiomes and plant communities in the field
Source: ISME Commun. 2022 Jul 26;2:59. doi: 10.1038/s43705-022-00144-1 (PMC9723724; doi:10.1038/s43705-022-00144-1)
Supplement: Supplementary file 1 — Supplementary Information [file 43705_2022_144_MOESM1_ESM.pdf]

Supplementary Information

Soil inoculum identity and rate jointly steer microbiomes and plant communities in the field

Xu Han[1, 2, 3 *], Yingbin Li[1 *], Yuhui Li[1, 3], Xiaofang Du[1], Bing Li[1, 3], Qi Li[1, a] and T. Martijn Bezemer[4, 5]

[1]*Erguna Forest-Steppe Ecotone Research Station, Institute of Applied Ecology, Chinese Academy of Sciences, Shenyang 110016, China.*

[2]*Forestry College, Beihua University, Jilin 132013, China*

[3]*University of Chinese Academy of Sciences, Beijing 100049, China.*

[4]*Department of Terrestrial Ecology, Netherlands Institute of Ecology (NIOO- KNAW), P.O. Box 50, Wageningen 6700 AB, Wageningen, The Netherlands.*

[5]*Institute of Biology, Above-Belowground Interactions group, Leiden University, P.O. Box 9505, 2300 RA Leiden, The Netherlands.*

[a] Corresponding author: Prof. Qi Li

Tel.: +86-24-83970359; fax: +86-24-83970300

E-mail address: liq@iae.ac.cn

[*] These authors contributed equally to this work.

This file includes Table S1, Figure S1, Figure S2, Figure S3, Figure S4 and Figure S5.

Table S1 Adonis test of the effect soil type, amount and their interactions on soil organism and plant communities in separate years.

		Year1			Year2			Year3		
		R^2	F	*P*	R^2	F	*P*	R^2	F	*P*
Bacteria	Soil	0.095	1.833	0.001	0.088	5.304	0.001	0.102	6.281	0.001
	Amount	0.155	1.504	0.002	0.07	2.108	0.001	0.072	2.214	0.001
	Soil*Amount	0.13	1.257	0.058	0.044	1.326	0.053	0.05	1.539	0.01
Fungi	Soil	0.095	1.77	0.001	0.078	4.518	0.001	0.081	4.719	0.001
	Amount	0.137	1.281	0.038	0.05	1.441	0.019	0.051	1.478	0.006
	Soil*Amount	0.125	1.169	0.126	0.043	1.257	0.081	0.04	1.17	0.118
Nematodes	Soil	0.025	1.305	0.151	0.058	3.239	0.001	0.134	8.438	0.001
	Amount	0.037	0.981	0.496	0.057	1.593	0.028	0.051	1.611	0.049
	Soil*Amount	0.025	0.67	0.92	0.029	0.8	0.779	0.052	1.637	0.051
Plants	Soil	0.193	15.463	0.001	0.107	7.258	0.001	0.097	5.958	0.001
	Amount	0.13	5.187	0.001	0.124	4.198	0.001	0.082	2.534	0.001
	Soil*Amount	0.078	3.119	0.001	0.061	2.071	0.008	0.042	1.305	0.157

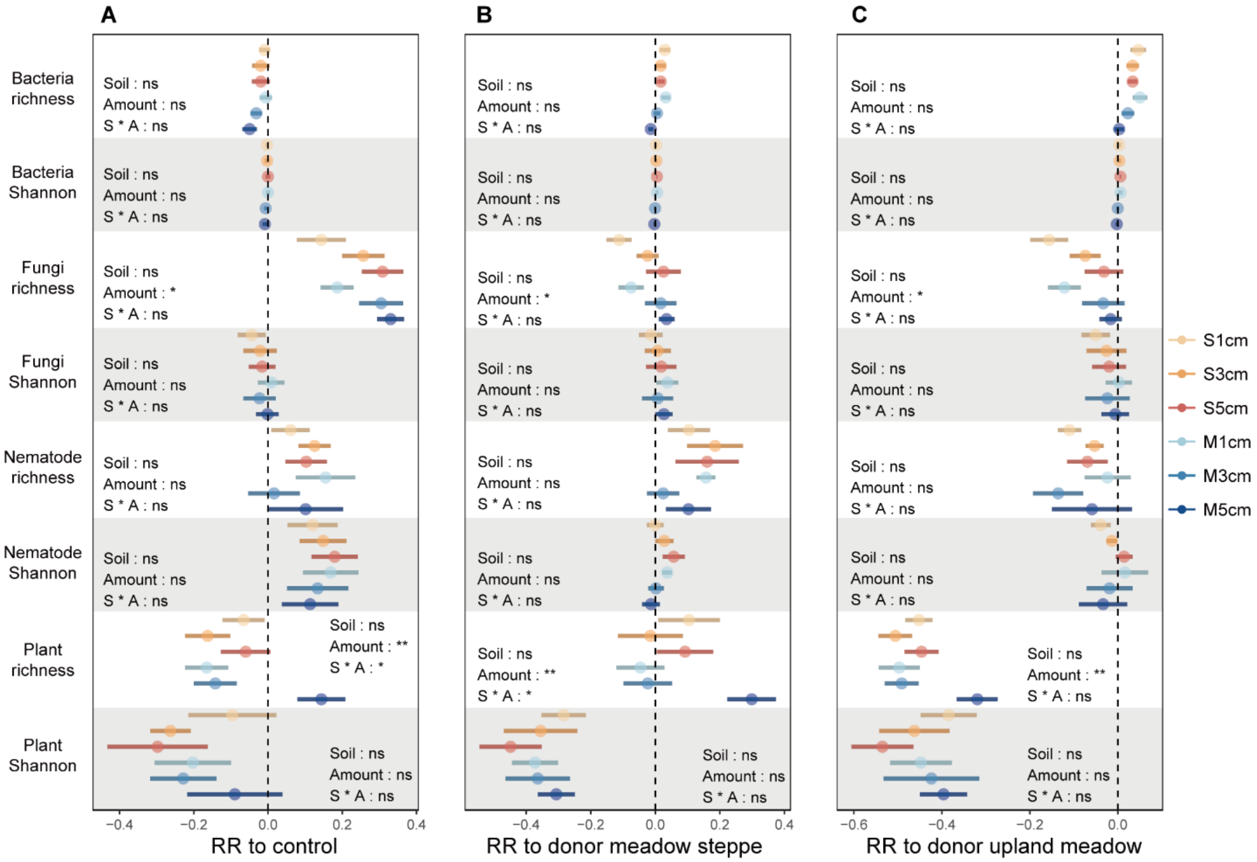

Fig. S1 Response ratio of alpha diversity of soil organism and plant communities to control, donor meadow steppe and donor upland meadow. Error bars represent ± S.E. The category definitions are as follows: S 1cm, meadow steppe soil inoculation with 1 cm depth; S 3cm, meadow steppe soil inoculation with 3 cm depth; S 5cm, meadow steppe soil inoculation with 5 cm depth; M 1cm, upland meadow soil inoculation with 1 cm depth; M 3cm, upland meadow soil inoculation with 3 cm depth; M 5cm, upland meadow soil inoculation with 5 cm depth. * $P < 0.05$; ** $P < 0.01$; *** $P < 0.001$.

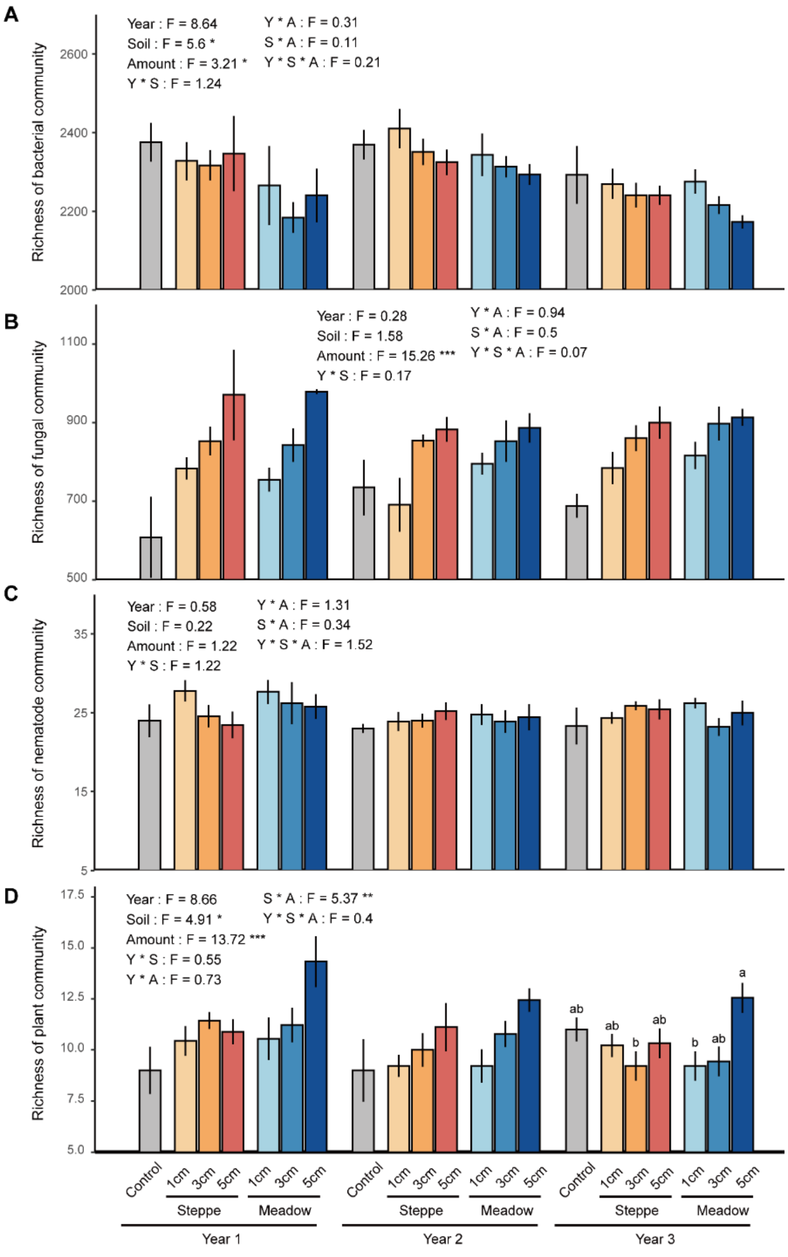

Fig. S2 Soil inoculation effect on the richness of bacterial, fungal, nematode and plant communities. The numbers at the top represent the result of linear mixed models. * $P < 0.05$; ** $P < 0.01$; *** $P < 0.001$. The category definitions are as follows: Control, the top soil removed treatment (TSR); Steppe, inoculation with meadow steppe soil; Meadow, inoculation with upland meadow soil. Letters in bars represent the difference within one year, different letters indicate significant differences at $P < 0.05$. Error bars represent ± S.E.

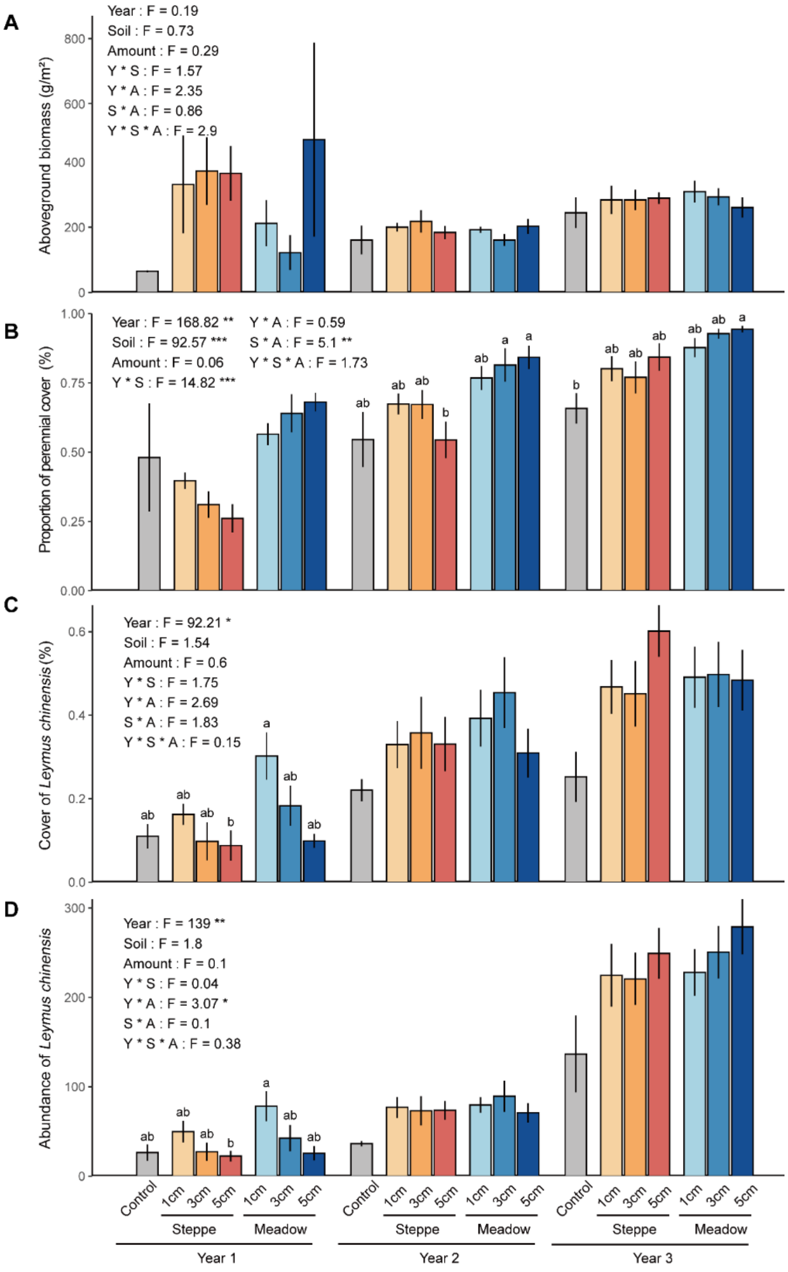

Fig. S3 Soil inoculation effect on plant traits in soil inoculation treatments. The numbers at the top represent the result of linear mixed models. * $P < 0.05$; ** $P < 0.01$; *** $P < 0.001$. The category definitions are as follows: Control, the top soil removed treatment (TSR); Steppe, inoculation with meadow steppe soil; Meadow, inoculation with upland meadow soil. Letters in bars represent the difference within one year, different letters indicate significant differences at $P < 0.05$. Error bars represent ± S.E.

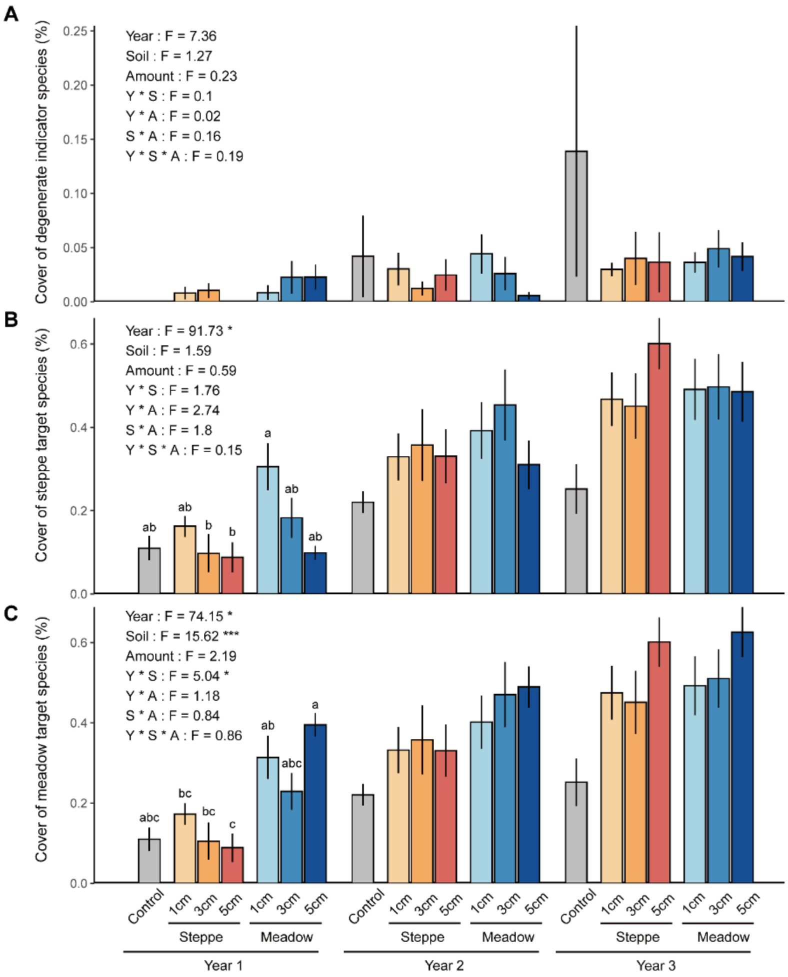

Fig. S4 Soil inoculation effect on the cover of degenerate indicator species, meadow steppe target species and upland meadow target species. The numbers at the top represent the result of linear mixed models. * $P < 0.05$; ** $P < 0.01$; *** $P < 0.001$. The category definitions are as follows: Control, the top soil removed treatment (TSR); Steppe, inoculation with meadow steppe soil; Meadow, inoculation with upland meadow soil. Letters in bars represent the difference within one year, different letters indicate significant differences at $P < 0.05$. Error bars represent ± S.E.

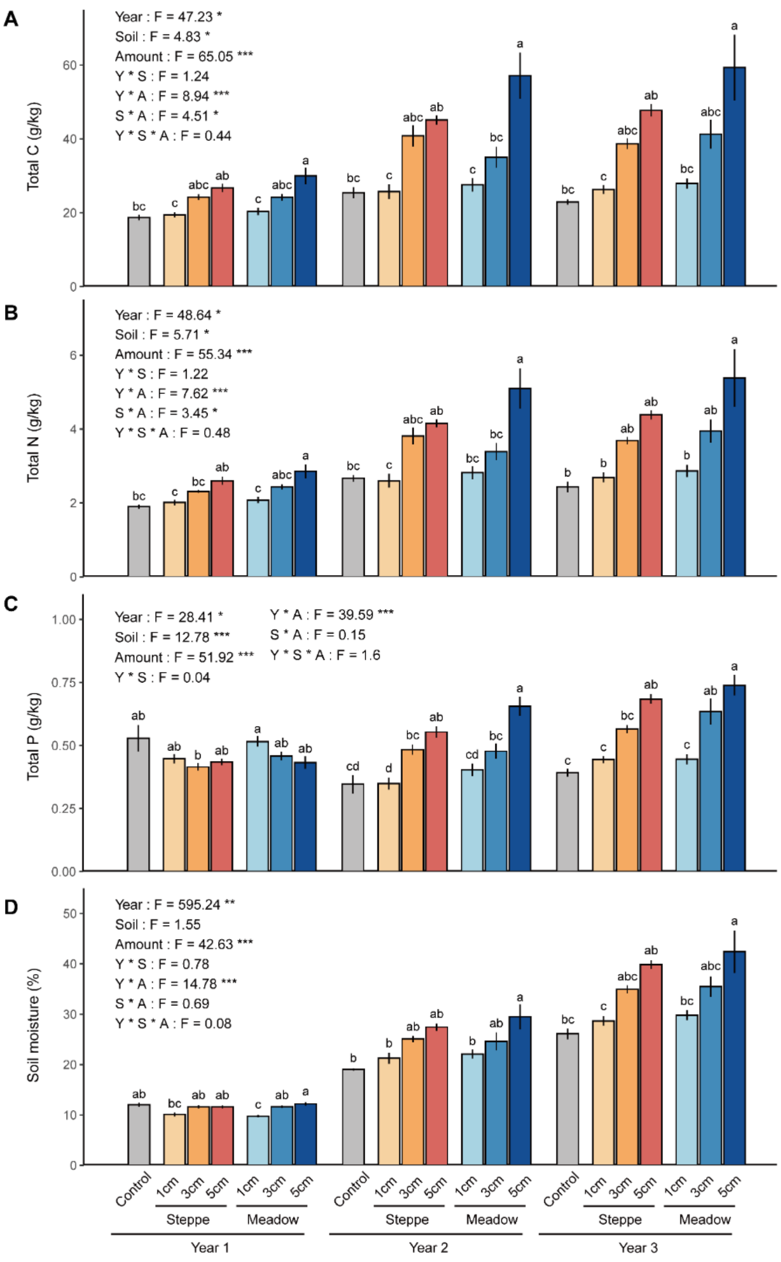

Fig. S5 Soil inoculation effect on soil nutrient and soil moisture. The numbers at the top represent the result of linear mixed models. * $P < 0.05$; ** $P < 0.01$; *** $P < 0.001$. The category definitions are as follows: Control, the top soil removed treatment (TSR); Steppe, inoculation with meadow steppe soil; Meadow, inoculation with upland meadow soil. Letters in bars represent the difference within one year, different letters indicate significant difference at $P < 0.05$. Error bars represent ± S.E.
